# The ICD-11 classification of personality disorders: a European perspective on challenges and opportunities

**DOI:** 10.1186/s40479-022-00182-0

**Published:** 2022-04-01

**Authors:** Bo Bach, Ueli Kramer, Stephan Doering, Ester di Giacomo, Joost Hutsebaut, Andres Kaera, Chiara De Panfilis, Christian Schmahl, Michaela Swales, Svenja Taubner, Babette Renneberg

**Affiliations:** 1 Center for Personality Disorder Research, Slagelse Psychiatric Hospital, Region Zealand, Slagelse, Denmark; 2 Institute of Psychotherapy/General Psychiatry, Lausanne University Hospital, Lausanne, Switzerland; 3 Department of Psychoanalysis and Psychotherapy, Medical University of Vienna, Vienna, Austria; 4 School of Medicine and Surgery, University of Milan Bicocca, Milan, Italy; 5 Viersprong Institute for Studies on Personality Disorders, Halsteren, Netherlands; 6 Kanta-Häme Central Hospital, Hämeenlinna, Finland; 7 Unit of Neuroscience, Department of Medicine and Surgery, University of Parma, Parma, Italy; 8 Department of Psychosomatic Medicine and Psychotherapy, Central Institute of Mental Health, Medical Faculty Mannheim, University of Heidelberg, Heidelberg, Germany; 9 School of Psychology, Bangor University, Bangor, UK; 10 Institute for Psychosocial Prevention, University Heidelberg, Heidelberg, Germany; 11 Clinical Psychology and Psychotherapy, Freie Universität, Habelschwerdter Allee 45, 14195 Berlin, Germany

**Keywords:** ICD-11, Avoidant personality disorder, Borderline personality disorder, Narcissistic personality disorder, Classification, Diagnosis, Personality trait, Severity

## Abstract

The 11th revision of the World Health Organization (WHO) International Classification of Diseases (ICD-11) includes a fundamentally new approach to Personality Disorders (PD). ICD-11 is expected to be implemented first in European countries before other WHO member states. The present paper provides an overview of this new ICD-11 model including PD severity classification, trait domain specifiers, and the additional borderline pattern specifier. We discuss the perceived challenges and opportunities of using the ICD-11 approach with particular focus on its continuity and discontinuity with familiar PD categories such as avoidant PD and narcissistic PD. The advent of the ICD-11 PD classification involves major changes for health care workers, researchers, administrators, and service providers as well as patients and families involved. The anticipated challenges and opportunities are put forward in terms of specific unanswered questions. It is our hope that these questions will stimulate further research and discussion among researchers and clinicians in the coming years.

## Introduction

All WHO member countries are expected to migrate from the ICD-10 to the ICD-11 Classification of Mental Disorders, which will be used for coding purposes, national statistics, and billing for health insurance companies [1]. This migration is particularly pertinent for European countries, which have been designated to be the first in this enterprise, starting from January 2022. While many diagnoses remain largely unchanged, a fundamentally different approach to classification of personality disorders (PD) is introduced in ICD-11 [2, 3]. Instead of diagnosing PDs according to familiar categorical types, the practitioner must now focus on general impairments of self- and interpersonal functioning, which can be classified according to their overall severity (i.e., *Mild Personality Disorder*, *Moderate Personality Disorder*, *Severe Personality Disorder*). Alternatively, the user can assign a sub-threshold *Personality Difficulty* code (comparable to ICD-10 Z73.1 accentuated personality traits). In order to further characterize individual features, the practitioner also has the option to specify one or more trait domain specifier that contribute to the individual expression of personality dysfunction. These trait domains are: *Negative Affectivity*, *Detachment*, *Dissociality*, *Disinhibition*, *Anankastia*. Finally, with the purpose of facilitating the identification of individuals who may respond to evidence-based treatments according to international and national guidelines [4], a *Borderline Pattern specifier* has been included, which is based on the DSM-5 Borderline PD diagnostic criteria. Given this dramatic shift in practice and research, we take the opportunity in this article to inform and describe the nature of this profound and paradigmatic change to a diagnostic system of personality disorder marked by the concepts of severity and trait domains, and highlight challenges and opportunities that arise in the discussion and implementation of these changes.

Before moving on, we would like to make two essential clarifications: First, the present paper exclusively relies on the now official ICD-11 PD model, which eventually was modified per 2018 in response to WHO’s dialogues with the European Society for the Study of Personality Disorders (ESSPD), the North American Society for the Study of Personality Disorders (NASSPD), and the International Society for the Study of Personality Disroders (ISSPD) [3, 5]. For that reason, we have omitted any reference to the ICD-11 PD model that predates 2018. Secondly, we exclusively refer to the Clinical Descriptions and Diagnostic Guidelines (CDDG) for Personality Disorders and Related Traits, which are aimed for clinical practice and can be accessed through the WHO Global Clinical Practice Network (gcp.network) or the complete ICD-11 browser version (icd.who.int). An abbreviated browser version also exists, which is only intended for statistical and coding purposes (e.g., insurance companies and secretaries).

### The function and importance of a diagnosis

The assignment of a diagnosis in mental health care may serve at least five purposes [6]: 1) conceptualization of psychopathology and presenting complaints, 2) communication of clinical information to practitioners, health care providers, administrators, researchers, and patients and their families, 3) differential diagnosis and assignment of the most relevant diagnosis, 4) informing treatment planning and effective interventions to improve clinical outcome, and 5) prediction of future treatment needs and expected magnitude of disability leave. For example, a transparent communication referring to diagnostic terminology often comprises the patient’s first encounter with mental health care. This is important, because the diagnostic description may instigate hope for change, lessen the sense of loneliness and alienation, and increase the feeling of being understood by a trusted expert [7]. Alternatively, a diagnosis may also create or increase stigma, which may be particularly problematic in adolescents who are still in the process of self-definition [8, 9]. At best, assigning a diagnosis may establish a foundation for a collaborative process moving towards reclaiming a life with less mental and interpersonal distress. In the light of the aforementioned functions of a diagnosis, it seems evident that a classification system that is overly complex or too simple, ill-understood or misinterpreted, misleading or misused may be of little practical use or even be harmful. At the same time, a diagnostic system with poor reliability and validity cannot inform accurate clinical prognosis or decision-making. Therefore, we want to transparently discuss and highlight anticipated challenges and opportunities related to the utility of the new ICD-11 classification of personality disorders.

## What is new in the ICD-11 classification of personality disorders?

In contrast to the established categorical approach to Personality Disorders (PDs) in ICD-10 and DSM-5 (i.e., 8–12 distinct types), the ICD-11 focuses on global and shared features that apply to *all* PDs as illustrated in Tables 1 and 2 [2]. These features comprise a substantial part of the general diagnostic requirements for the presence of a PD. Accordingly, the practitioner or researcher must diagnose a PD based on a global evaluation of self- and interpersonal functioning, cognitive, emotional, and behavioral manifestations, and psychosocial impairment and distress [2].

**Table 1:** Aspects of personality functioning that contribute to severity determination in Personality Disorder

● **Degree and pervasiveness of disturbances in functioning of aspects of the self**	
○ Stability and coherence of one’s sense of identity (e.g., extent to which identity or sense of self is variable and inconsistent or overly rigid and fixed). ○ Ability to maintain an overall positive and stable sense of self-worth. ○ Accuracy of one’s view of one’s characteristics, strengths, limitations. ○ Capacity for self-direction (ability to plan, choose, and implement appropriate goals).	
● **Degree and pervasiveness of interpersonal dysfunction across various contexts and relationships (e.g., romantic relationships, school/work, parent-child, family, friendships, peer contexts).**	
○ Interest in engaging in relationships with others. ○ Ability to understand and appreciate others’ perspectives. ○ Ability to develop and maintain close and mutually satisfying relationships. ○ Ability to manage conflict in relationships.	
● **Pervasiveness, severity, and chronicity of emotional, cognitive, and behavioural manifestations of the personality dysfunction** ○ Tendency to be emotionally over- or underreactive, and having difficulty recognizing unwanted emotions (e.g., does not acknowledge experiencing anger or sadness) ○ Distortions in the accuracy of situational and interpersonal appraisals under stress (e.g., dissociative states, psychotic-like beliefs or perceptions, and paranoid reactions). ○ Behavioural responses to intense emotions and stressful circumstances (e.g., propensity to self-harm or violence).	
● **The extent to which the dysfunctions in the above areas are associated with distress or impairment in personal, family, social, educational, occupational or other important areas of functioning.**	

Note. This abbreviated content is adapted from WHO ICD-11 Clinical Descriptions and Diagnostic Guidelines for Mental and Behavioural Disorders [2]. The listed features and examples are not exhaustive

**Table 2:** Overview of the ICD-11 Classification of Personality Disorders

*Level of Personality Dysfunction*	
None	
Personality Difficulty	
Mild PD	
Moderate PD	
Severe PD	
*Trait Domain Specifiers*	
Negative Affectivity	
Detachment	
Disinhibition	
Dissociality	
Anankastia	
*Borderline Pattern Specifier (if applicable)*	

Note. The dashed line represents the threshold for a PD diagnosis. As evident, the diagnostic threshold is not between PD and “None”, but between PD and sub-diagnostic personality difficulty

### Classification of severity: what level of personality disorder?

After establishing the presence of a PD, the practitioner may determine whether the patient’s level of personality problems overall corresponds to a *Mild Personality Disorder*, a *Moderate Personality Disorder*, or a *Severe Personality Disorder*. For example, some patients’ sense of self may only be contradictory or inconsistent (*Mild Personality Disorder*), while other patients have a highly unstable or internally contradictory sense of self (*Severe Personality Disorder*). Likewise, the patient’s situational and interpersonal appraisals may in certain cases involve some distortions but with intact reality testing (i.e., *Mild Personality Disorder*), while other patients experience extreme distortions under stress often including dissociative states or psychotic-like perceptions and interpretations (i.e., *Severe Personality Disorder*). As a final example, the ICD-11 classification of PD severity also incorporates harm to self and others, where patients with milder PD cause no significant harm while patients with severe PD often cause severe harm (e.g., repetitive self-injurious or aggressive behaviors).

The ICD-11 provides a list of essential features for each of the three categories of severity (i.e., mild, moderate, severe), which are accompanied by a list of examples that may guide practitioners in their decision-making. It is important to underscore that these features and examples should not be used as diagnostic “criteria”, but they should only be used as guidelines for a more global evaluation. Moreover, as will be discussed later in the present paper, the dimensional conception of PD severity is virtually being translated into ordered categories of severity.

### The option of coding sub-diagnostic personality difficulty

In addition to the PD diagnosis (at least “Mild Personality Disorder”), there is an option to assign a sub-diagnostic code for the presence of *Personality Difficulty*. Personality Difficulty is not a disorder per se, but is available as a code to inform treatment and preventive care, and is located in the section of the ICD-11 classification for non-disease entities that constitute factors influencing health status and encounters with health services. Thus, Personality Difficulty can be compared to the ICD-10 non-disorder codes for “accentuation of personality traits” (Z73.1) or “borderline intellectual functioning” (R41.83). This code may typically be used in cases where there is an issue with personality that must be addressed (e.g., perfectionism or anxiousness) or to recognize that a patient, who has undergone successful treatment of a PD, still has some residual features of the personality disturbance, which other health professionals should pay attention to. In contrast to a Personality Disorder diagnosis, *Personality Difficulty* is typically less complex and only limited to specific situations or relationships. Problems typically occur with less intensity or are only expressed intermittently (e.g., during times of stress and pressure).

### Trait domain specifiers: what kind of personality disorder problems?

In addition to the coding of severity, more detailed specifier codes are available. Given that personality functioning might be impaired in different ways, the trait domain specifiers are available to characterize the specific pattern of traits (i.e., style) that contribute to the global personality dysfunction. These specifiers serve to describe the individual trait expressions of the personality disturbance (i.e., the flavor of the disorder). For example, it makes a substantial difference whether the impairment is related to being overly anxious and avoidant (e.g., Negative Affectivity and Detachment) or very self-centered and dominant (e.g., Dissociality). Those two different trait expressions reflect different kinds of problems and may inform different treatment approaches.

It is possible to assign as many trait specifiers as necessary to describe the individual. Interpretation of different trait domain combinations tends to say more about the person than interpretations at the individual domain level. For example, two persons characterized by Negative Affectivity may evidently share certain features of this trait domain. However, the first person has a combination with Detachment (e.g., internalized anger and self-blaming), whereas the other has a combination with Dissociality (e.g., externalized anger and blaming others). Moreover, the number or complexity of trait domain specifiers often mirrors the global severity. Thus, a severe PD is likely to be associated with several trait domain specifiers, whereas a Mild PD may be associated with the presence of only one trait specifier. However, in some cases a patient may have a severe PD and manifest only one prominent trait specifier (e.g., Dissociality causing severe danger towards others). On the one hand, this basic classification of PD severity may be viewed as too simplistic and uninformative in comparison to the larger number of categories and criteria presented in the ICD-10. On the other hand, based on the potential number of ICD-11 trait combinations, the practitioner is allowed to portray 31 different trait patterns with at least one trait specifier for each case. Thus, at the most sophisticated level, we may also take all three severity categories plus 31 trait compositions into account, which virtually allows clinicians to describe 93 possible variations of a PD diagnosis. Moreover, the facet-like features of the trait domains (e.g., emotional lability, mistrust, entitled superiority) may also be operationalized using various subscales [10]. See Table 3 for a comparative overview of strengths and weaknesses across the ICD-10 and ICD-11 models.

**Table 3:** Strengths and Weaknesses of the ICD-10 and ICD-11 models of Personality Disorders

*Strengths*	*Weaknesses*
***ICD-10 Classification of Personality Disorder types***	
Are based on a well-established and longstanding tradition of clinical observations.	Suffer from heterogeneity and excessive co-occurrence (e.g., most patients meet criteria for at least one other category).
Clinicians tend to think in terms of types or “gestalts”.	Clinicians tend only to use the categories of Borderline, Antisocial, and Unspecified Personality Disorder, while neglecting the other categories.
Polythetic criteria allow many different combinations and variations of Personality Disorder types.	Two different patients with the same Personality Disorder type may not share a single symptom (e.g., Schizoid), which allows unclear diagnostic patterns.
Are largely consistent with established clinical theory, and have been subjected to extensive research.	There is limited evidence (with the exception of Borderline-related features) that Personality Disorder types are sound phenotypes or biological markers.
Categorical diagnostic thresholds match categorical decision-making in medical practice and requirements by insurance companies.	Diagnostic thresholds may be pseudo-accurate and clinical decision-making is not always a categorical matter of “present” versus “absent”, and subthreshold diagnosis may have clinical significance.
Provides a manageable number of personality disorder categories (i.e., 8–10 types).	The polythetic categorical approach includes 58 specific criteria in addition to 6 general diagnostic criteria, which can be cumbersome for busy practitioners to evaluate.
***ICD-11 classification of severity and trait domains***	
A global severity determination informs prognosis, risk, and intensity of treatment.	A global severity determination, without considering typology, may be vague, imprecise, and therefore not very informative.
A global severity classification is simple and manageable for low resource settings, and it prevents diagnostic co-occurrence.	A global severity classification may be too minimalistic and unsophisticated for specialist clinical practice.
The option of portraying compositions of 3 severity levels and 5 additional trait domains virtually allows clinicians to describe 93 variations of a personality disorder.	A total of 93 different compositions of a personality disorder diagnosis can be too complex for clinical practice and communication.
Trait domains are empirically-derived “building blocks” of personality pathology.	Many clinicians are unfamiliar with the trait domains - and it is not straightforward how to translate them into clinical practice.
Classification of severity and trait domains allow future treatment trials to focus on global human functioning as well as homogenous phenotypes (i.e., trait domains).	No longer correspondence with established research and clinical recommendations for personality disorder types (except for Borderline).
Continuity with empirical taxonomies of a global p-factor, internalizing-externalizing spectra, the five-factor model, and the DSM-5 Alternative Model of Personality Disorders (AMPD).	Discontinuity with familiar, well-established, and historically important personality disorder types (except for Borderline).

### Borderline pattern specifier

In addition to the classification of PD severity and the most prominent trait domains, the ICD-11 also provides a borderline pattern specifier, which essentially relies on DSM-IV/5’s definition of Borderline PD (see link). Thus, in contrast to the ICD-10 operationalization of F60.3 Emotionally unstable PD (i.e., F60.30 impulsive subtype and F60.31 borderline subtype), the ICD-11 Borderline Pattern specifier is defined by the nine familiar DSM-IV/5 features including “dissociative symptoms or psychotic-like features (e.g., brief hallucinations, paranoia in situations of high affective arousal)”. In supplement to these nine features, the user may also take three additional manifestations of the Borderline Pattern into consideration, which may be of help for both diagnostic pattern recognition, more fine-grained clinical description, and treatment planning: 1) a view of self as bad, inadequate, guilty, and contemptible [11]; 2) a sense of alienation or loneliness [12]; and 3) rejection sensitivity, problems with trust, and misinterpretation of social signals [13, 14].

Despite a large body of treatment evidence and recognition by health authorities, the initial ICD-11 proposal did not distinguish the presence of a Borderline PD type because of its debated construct validity. In response, representatives from the European Society for the Study of Personality Disorders (ESSPD), among others, highlighted the need to include a “borderline pattern specifier” in the ICD-11 classification [15]. Thus, the eventual inclusion of this specifier reflects a pragmatic compromise between divergent positions [5]. The challenge was how to move to a fundamentally new classification system without abandoning the observed strengths of the previous one, including its capacity to capture the manifestations of Borderline PD. In this respect, the official inclusion of a borderline pattern specifier ensures continuity and clinical utility of the ICD-11 system for all practitioners, researchers, and patients by facilitating the identification of individuals who may respond to well-established evidence-based treatments [16, 17].

#### What about “co-morbidity” among personality disorders?

The ICD-11 PD model addresses the issue of co-occurrence or comorbidity of disorders by requesting from the clinician to assess the severity of the PD itself, rather than focusing on heterogenous categories. The three severity levels, by definition, cannot co-exist with one another within the same patient at the same time. Thus, by focusing on global PD features, the ICD-11 classification can be said to bypass the within-group co-morbidity that characterizes ICD-10 PD categories. Nevertheless, the composition of individual personality styles may be characterized using the non-diagnostic trait specifiers that naturally co-occur with one another. The ICD-11 PD guidelines explicitly provide this instruction: “As many trait domain specifiers may be applied as necessary to describe personality functioning” [2].

By serving as homogenous “building blocks” of personality pathology, the trait domain specifiers help elucidate and disentangle the overlapping features we know from the PD categories [18]. For example, Negative Affectivity applies to both Dependent PD (“preoccupation with fears of being abandoned by a person with whom one has a close relationship”) and Borderline PD (“excessive efforts to avoid abandonment”). In a similar manner, Negative Affectivity also applies to both Anankastic PD (“feelings of excessive doubt and caution”) and Avoidant PD (“restrictions in lifestyle because of need to have physical security”). The trait domains of Dissociality and Disinhibition are equally involved in Dissocial PD (“very low tolerance to frustration and a low threshold for discharge of aggression, including violence”) and Borderline PD (“liability to outbursts of anger or violence, with inability to control the resulting behavioural explosions”). Likewise, we also see certain features of Dissociality in both Paranoid PD (“a combative and tenacious sense of self-righteousness out of keeping with the actual situation”), Anankastic PD (“unreasonable insistence that others submit to exactly his or her way of doing things”), and Borderline PD (“marked tendency to quarrelsome behaviour and to conflicts with others, especially when criticized”).

#### Retaining “categories” of severity and trait domains

Like with the DSM-5 Alternative Model of Personality Disorders (AMPD) model, the ICD-11 approach has commonly been branded as a “dimensional” model [19]. Such emphasis on dimensions has been a major concern to some clinicians who prefer categories to facilitate clinical decision-making and treatment planning [15, 20]. Nevertheless, while ICD-11 recognizes that PD severity and traits are dimensional in nature, the diagnostic codes can only be assigned as if they were categories ranked on an ordinal scale. Accordingly, the clinical practitioner first of all has to decide whether or not the patient has a PD based on the general diagnostic requirements, which is essentially a categorical decision. Subsequently, the practitioner has to determine whether the patient has a *Mild Personality Disorder*, a *Moderate Personality Disorder*, or a *Severe Personality Disorder*, which also correspond to a categorical decision. This is in fact substantially comparable to the diagnosis of a depressive episode (i.e., mild, moderate, severe). In a similar way, the most prominent ICD-11 PD trait specifiers can also only be coded as present or absent even though they exist on a continuum. So, are we actually dealing with PD categories? The answer is both “yes” and “no”. From a scientific perspective, the constructs are dimensional (e.g., there is no true threshold between “mild” and “moderate”), while in clinical practice they must be operationalized as categorical codes ranked on an ordinal scale. In other words, research may continue focusing on the continuum of personality disturbances and trait domains (including sub-diagnostic levels), while practitioners may directly translate findings into the diagnostic categories for the purpose of clinical decision-making. As a future prospect, dimensional measurement of PD severity can be converted into categories by the incorporation of cut-off points with significance for risk, impairment, treatment, and prognosis. Such data is not yet available apart from a preliminary cut-score proposed for the Personality Disorder Severity ICD-11 (PDS-ICD-11) scale [21].

#### Available instruments for the ICD-11 classification of personality disorders

Essentially, practitioners in WHO member countries should be able to diagnose a PD using the available ICD-11 Clinical Descriptions and Diagnostic Guidelines [2] per se without having to use additional measures or instruments. Therefore, it should be possible for clinicians to determine PD severity based on clinical observations and other available information. Meanwhile, standardized instruments are necessary for ensuring sufficient reliability. Instruments developed for the DSM-5 AMPD model may also be helpful in the classification of ICD-11 PD severity and trait domains. Nevertheless, it is preferred to have a standardized tool to operationalize the specific ICD-11 diagnostic PD features including the particular cognitive (e.g., reality testing), emotional (e.g., being over- or underreactive), and behavioral (e.g., harm to self or others) manifestations that exclusively apply to the ICD-11 approach. To fill this gap, patient-report scales have recently been published [10, 21–23] and a formal diagnostic interview is under development and will be made available for practitioners and researchers. A preliminary study found that ICD-11 PD severity can be diagnosed with excellent inter-rater reliability (intra-class correlation coefficient of .95 [95% CI = 0.89, 0.98 *p* < 0.001]) based on 2 raters who assessed 20 patients according to information from a STiP 5.1 interview [21]. However, more studies investigating the inter-rater reliability of a structured interview for ICD-11 PD severity are warranted. We provide an overview of currently available instruments in Table 4, which have proven to be promising to address this gap.

**Table 4:** Instruments for the operationalization of ICD-11 personality disorder diagnosis

*Personality Disorder Severity*	*Trait Domain Specifiers*
• Personality Disorder Severity ICD-11 (PDS-ICD-11) scale [21] – 14 items • Level of Personality Functioning Scale – Brief Form (LPFS-BF) [24] - 12 items • Self- and Interpersonal Functioning Scale (SIFS) [25] – 24 items • Level of Personality Functioning Questionnaire – 12-18 (LoPF-Q-12-18) for use with adolescents [26] – 97 items • Scales for ICD-11 Personality Disorder: Self and Interpersonal Dysfunction [10] – 65 items • Structured Clinical Interview for DSM-5 Alternative Model of Personality Disorders (SCID-AMPD) Module I [27] • Semi-structured interview for Personality Functioning DSM-5 (STiP 5.1) [28]	• Personality Inventory for ICD-11 (PiCD) [29] delineating 5 domains – 60 items • Five-Factor Inventory for ICD-11 (FFiCD) [22] including 5 domains, 20 facets, and 47 nuances – 121 items • Personality Inventory for DSM-5 (PID-5) with ICD-11 algorithm [18, 30] – 158 items • Personality Inventory for DSM-5 and ICD-11 Plus Modified (PID5BF + M) [31, 32] – 36 items • Personality Assessment Questionnaire for ICD-11 personality trait domains (PAQ-11) [23] – 17 items • Scales for ICD-11 Personality Disorder: Five Personality Disorder Trait Domains [10] – 181 items • Structured Clinical Interview for DSM-5 Alternative Model of Personality Disorders (SCID-AMPD) Module II [33] with ICD-11 algorithm

## How to translate the ICD-10 categories into the new ICD-11 classification?

To facilitate some continuity with established clinical practice, it seems important to understand the new ICD-11 approach with reference to the traditional ICD-10 PD types. We are entering a transition phase in which both systems might be used side by side in order to gain the necessary knowledge about this question. It is important to underscore that such transition should be limited to a certain period of time as we eventually must leave the ICD-10 PD categories behind and entirely commit to the official ICD-11 system as a standalone approach.

Some of the familiar PD types described in ICD-10 seem fairly recognizable in the ICD-11 trait domain specifiers. For example, it appears straightforward that the trait domain of Anankastia resembles the Anankastic or Obsessive-Compulsive PD. Likewise, the trait domain of Dissociality resembles the Dissocial PD, while the trait domain of Detachment resembles the Schizoid PD. However, the very common and frequently discussed types of Avoidant PD (ICD-10 F60.6 Anxious personality disorder) and Narcissistic PD (ICD-10 F60.8 Other specific personality disorder) seem less straightforward to characterize using trait domain specifiers. This might be critical as Avoidant PD is highly prevalent and associated with poor psychosocial functioning but often goes unrecognized. At the same time there are empirically supported treatment approaches available [34, 35]. In clinical contexts, Narcissistic PD often poses a challenge for treatment [36]. In the following, we therefore seek to portray these two familiar types using specific ICD-11 PD definitions.

### Characterizing avoidant personality disorder within the new system

It is assumed that individuals with Avoidant PD may virtually be characterized by features ranging from mild to severe PD. They are characterized by marked difficulty in self-esteem along with intense fear of criticism and rejection. Their ability to work towards goals is often compromised due to lack of self-confidence and anxiousness. Likewise, their relationships are characterized by avoidance, which compromises social and occupational roles. Individuals with this pattern typically do not cause substantial harm to others, but may cause harm to themselves.

According to meta-analytic evidence, Avoidant PD is generally characterized by a combination of Negative Affectivity and Detachment [37, 38]. With respect to Negative Affectivity, this pattern particularly involves anxiousness, shame, low self-esteem, and a tendency to be over-reactive to external events (e.g., perceived threats of criticism or potential future problems). These patients’ low self-esteem and lack in self-confidence manifest in terms of *avoidance of situations and activities* that are perceived as too difficult (e.g., intellectually, physically, socially, interpersonally, emotionally, etc.), even despite evidence to the contrary.

With respect to Detachment, the Avoidant PD pattern involves social Detachment characterized by avoidance of social interactions, lack of friendships, and avoidance of intimacy. Due to anxiousness and low self-esteem, they either avoid social interactions completely or endure them with extreme discomfort. These patients tend to engage in little to no ‘small talk’ even if initiated by others (e.g., at store check-out counters), seek out employment that does not involve interactions with others, and even refuse promotions if it would entail more interaction with others. The complete Avoidant PD pattern of Negative Affectivity and Detachment is overall consistent with the description of Avoidant PD patients as being both fearful and emotionally inhibited [39–42]. In addition, Avoidant PD features may also be illuminated by scales developed to capture ICD-11 trait facets and nuances such as evaluation apprehension, social isolation, shame, interpersonal inadequacy, unassertiveness, risk aversiveness, ineptitude, and fragility (see Table 4) [10, 22, 31].

### Characterizing narcissistic personality disorder within the new system

Individuals with a Narcissistic PD may be characterized by features ranging from mild to severe PD [2]. Their self-view can vacillate between overly positive and omnipotent, and extraordinarily negative and devastating. Depending on the specific nature of the narcissistic disorder (i.e., grandiose or vulnerable), such individuals may have difficulty recovering from injuries to their grandiose and vulnerable self-image. They may exhibit poor emotion regulation in the face of setbacks. Their self-focus and callousness may compromise the quality of their relationships, in particular by ignoring other’s opinions or exploiting others, which may contribute to their difficulties in developing close and mutually satisfying relationships. Their existing relationships may often be characterized by volatile and one-sided conflicts, where they may appear as strongly dominant. For the same reason, a sub-group of these patients may be unable to sustain regular work conditions or collaboration.

Narcissistic features are essentially characterized by the trait domain of Dissociality with emphasis on self-centeredness [18]. This pattern involves a sense of exploitativeness of others, believing and acting as if they deserve whatever they want which, in their eyes, should be obvious to others. Such features of narcissism can be manifested as an expectation of others’ admiration, attention-seeking behaviours to ensure being the center of others’ focus, and anger or denigration of others when the admiration and attention that the individual expects are not granted. Typically, such individuals believe that their accomplishments are outstanding, that they have many admirable qualities, that they have or will achieve greatness, and that others should admire them.

As an anticipated challenge for clinicians, the Dissociality trait domain specifier may not appear very specific for narcissism because it would also characterize dissocial PD and psychopathy. Nevertheless, many individuals with the diagnosis of a Narcissistic PD, in order to keep up with a subjective sense of superiority, are also characterized by the trait domain of Anankastia in terms of perfectionism and vanity, which serves to enhance competitiveness, self-esteem, and grandiose self-presentations [43]. Accordingly, the combination of Dissociality and Anankastia may often indicate distinct features of narcissism, including perfectionistic overcompensation and rule-bound narcissistic dominance. Likewise, additional features of Negative Affectivity in terms of vulnerability, depression, anger, hostility, and shame may also capture vulnerable manifestations of narcissism. Thus, the combination of Dissociality and Negative Affectivity may characterize some individuals with vulnerable narcissism who are ruminating over perceived slights or insults from others, are overreactive to criticism, and have a low frustration tolerance that easily makes them become overtly or covertly upset over even minor issues. Their low self-esteem may manifest as envy of others’ abilities and success, and it may also be driven by shameful experiences of repeated failures and procrastinations in their lives. Taken together, individual manifestations of narcissism may be captured by distinctive combinations of trait domains where Dissociality serves as the main ingredient. In addition, narcissistic features may also be illuminated by scales developed to capture ICD-11 trait facets and nuances such as grandiosity, need for admiration, vanity, arrogance, selfishness, reactive anger, shame, self-centeredness, lack of empathy, and entitled superiority (see Table 4) [10, 22, 31].

### Where do we go from here?

The advent of the ICD-11 classification of PDs will be a significant change for health care workers, psychotherapists, researchers, insurance companies, administrators, and service providers, as well as patients and families concerned. When discussing how to proceed from here, we must first and foremost acknowledge that much is still unknown. Accordingly, in the following we aim to share with the readers a number of unanswered questions related to the perceived challenges and opportunities. We expect these questions to be further discussed and studied in the coming years, and that such endeavor will also help identify yet unknown obstacles, problems and opportunities.

### Diagnostic reliability

Even though development of specific semi-structured interview instruments are underway, there are no default instruments for the ICD-11 PD classification. This is consistent with WHO’s rationale that the diagnostic guidelines per se should be sufficient to make a diagnosis in clinical practice. While this may be an advantage for worldwide clinical utility and feasibility, it may prove to be a disadvantage for diagnostic reliability (e.g., inter-rater reliability) and research. For example, what we gain in validity, we may lose in diagnostic precision or reliability? For example, some clinicians may find it challenging to determine the overall severity-level when the patient may be characterized by features of more than one level of PD severity. We therefore propose that rigorous field trials should be conducted to determine the reliability of the diagnostic guidelines, with reference to the reliability of other established and well-validated instruments (e.g., SCID-5-PD). Table 4 provides an overview of instruments and measures, which are officially being recommended to be used for empirical investigations and clinical operationalization of the ICD-11 classification in the years to come.

### User preference investigations

Apart from the somewhat promising results of initial user preference studies [44, 45], it still remains inconclusive whether the ICD-11 PD classification truly fulfills the criteria for clinical utility. We therefore recommend that more studies are conducted with practitioners who are allowed to express what they think about this new system with respect to its perceived usefulness in routine clinical practice, and how it might be further improved in future revisions. Moreover, it would be valuable to include patients and their families in such surveys in order to get a service user perspective on the changes [45, 46]. Finally, it seems particular relevant to further investigate whether practitioners find the severity classification and the trait domain specifiers helpful for case formulation, treatment planning, and intervention as suggested by initial research [47, 48].

### Future relevance of the borderline pattern specifier

We welcome the inclusion of a borderline pattern specifier as a pragmatic solution to divergent positions and even more important to ensure access for patients to evidence-based treatments. Large-scale studies in different countries evaluating the utility of this specifier with reference to the established instruments for this construct are needed. Preliminary research suggests that global severity of personality dysfunction accounts for substantial parts of the variance described by Borderline PD [49–51]. Certain clinical research and meta-analytic evidence suggest that the heterogeneity of Borderline PD features may be captured by trait domains of Negative Affectivity and Disinhibition, along with some features of Dissociality [18, 37], although it seems clear that these three trait domains alone do not capture all possible borderline symptoms in a clinically relevant manner. For example, Borderline-related disturbances of identity and reality testing are only globally captured in the ICD-11 classification of PD severity, but not by any trait domain.

While the aforementioned findings do not question the clinical relevance of the borderline pattern specifier as a specific “pattern”, more research is needed on the relationships between the severity levels and trait domains on the one hand and the borderline pattern on the other [52]. Given the substantial burden of disease associated with Borderline PD, such research may become important in order to elucidate severity and trait patterns of Borderline PD, and help allocate resources effectively according to different treatment options that have already proven to be effective for Borderline PD. On the one hand, because of the demonstrated responsiveness to evidence-based treatments, the ICD-11 borderline pattern may in some cases become a frontrunner specifier and thereby encapsulating some of the challenges and opportunities with respect to established treatment programs [16]. On the other hand, ICD-11’s global PD severity classification and trait domain specifiers may appeal to the increased use of transdiagnostic and personalized treatments cutting across traditional categorical PD diagnoses [53].

### Is negative affectivity just a “One Size Fits All” trait specifier?

Some evidence suggests that the trait domain of Negative Affectivity explains a large amount of personality pathology observed in mental health services [18, 54]. However, it must be combined with other relevant trait domain specifiers in order to be more informative from a clinical viewpoint [18]. Metaphorically, Negative Affectivity may be compared to the juice that serves as base ingredient in many different cocktails. However, while such “cocktails” of trait domains may be clinically relevant, it remains questionable whether they actually capture the content of the familiar PD types that we usually treat.

From a critical perspective, the base ingredient of Negative Affectivity may potentially become a reductionistic “one size fits all” conceptualization of most PD cases. We therefore welcome exploratory work on how trait domains interact with one another at different levels of severity, and in particular how they are related to familiar ICD-10 PD types. The descriptions in the ICD-11 classification already convey a more complex understanding of Negative Affectivity. For example, it is highlighted that a combination with Detachment may cause self-blaming, whereas a combination with Dissociality may involve blaming of others.

The ICD-11 guidelines explicitly recognize that individuals with Negative Affectivity may exhibit poor self-esteem in a number of ways depending on context and other dynamics: a) avoidance of situations that are judged too difficult; b) dependency on others for advice, direction, and help; c) envy of others’ abilities and indicators of success; and d) suicidal ideations due to believing themselves to be useless. All four manifestations may even apply to the same individual across time and situation, all depending on context, complexity, and PD severity. The key message here is that the aforementioned patterns comprise different situational expressions of Negative Affectivity resulting in different implications for treatment. Taken together, Negative Affectivity per se may be understood as a “one size fits all” domain that applies to all emotional disorders, and when combined with other information, it may also reveal more clinically informative material. To further uncover such trait- and severity dynamics, it seems worthwhile to operationalize, portray, and investigate more fine-grained features (i.e., facets) of Negative Affectivity. Accordingly, facet-level scales have been developed to capture Negative Affectivity features such as emotional lability, negativistic attitudes, low self-esteem, and mistrustfulness (see Table 4) [10, 22, 31].

It remains an open question whether the concept of Negative Affectivity – alone or in combination with other domains – informs an explanatory formulation of the problems observed in a particular patient. Thus, disorder-specific case formulation methods may assist the clinician in this task, moving into the idiosyncratic details of each case. Problems associated with Negative Affectivity may be best explained by case formulation methodologies according to Dialectical-Behavior Therapy, the Unified Protocol, or Emotion-Focused Therapy [55, 56].

### Utility of severity classification for treatment decisions

The introduction of the ICD-11 classification of PD severity may help in clinical decision making and allocation of treatment resources (e.g., type, length, and intensity of treatment) [47]. More research is also needed to determine empirically-informed thresholds and the prognostic value of grouping patients into categories of severity.

However, some practitioners may also be concerned that certain severity levels can fall under insurance companies’ and service providers’ criteria for support due to financial constraints in many European countries. For example, insurance companies or service providers may decide only to cover treatment for those with a severe PD while neglecting those with milder forms. At worst, a simple ordinal scale intended for transparent use by clinicians may well turn into a political instrument of resource allocation in health systems or hospitals. At best, such approach to allocation of resources may actually help ensure treatment for those who need it the most rather than exclusively basing such decisions on individual practitioners’ private opinions and observations. In any case, it would be helpful if such a PD severity classification could improve early detection, prognostic evaluation, and targeted treatment of mild, moderate, and severe PDs.

## Conclusion

The advent of the ICD-11 classification for personality disorders will affect researchers, clinicians, patients, and their families. We have outlined several areas of potential challenges as well as potential opportunities of this new approach. Hopefully the challenges ahead will be addressed constructively by new lines of research, including the study of interactions among severity levels, trait domains, and the borderline pattern. The development of reliable assessment instruments and procedures also seems vital for our field. Finally, we need development of tailored treatment, which is informed by severity classification and prominent trait domain specifiers, including the opportunity to evaluate the effect of intervention according to the continuum of global PD severity.

## Data Availability

n.a.

## References

[1] Reed GM, First MB, Kogan CS, Hyman SE, Gureje O, Gaebel W (2019). Innovations and changes in the ICD-11 classification of mental, behavioural and neurodevelopmental disorders. World Psychiatry.

[2] WHO (2022). ICD-11 Clinical Descriptions and Diagnostic Guidelines for Mental and Behavioural Disorders.

[3] Reed GM (2018). Progress in developing a classification of personality disorders for ICD-11. World Psychiatry.

[4] Simonsen S, Bateman A, Bohus M, Dalewijk HJ, Doering S, Kaera A (2019). European guidelines for personality disorders: past, present and future. Borderline Personal Disord Emot Dysregul.

[5] Huprich SK, Herpertz SC, Bohus M, Chanen A, Goodman M, Mehlum L (2018). Comment on Hopwood et al., the time has come for dimensional personality disorder diagnosis. Personal Ment Health.

[6] First MB, Pincus HA, Levine JB, Williams JBW, Ustun B, Peele R (2004). Clinical utility as a criterion for revising psychiatric diagnoses. Am J Psychiatry.

[7] Perkins A, Ridler J, Browes D, Peryer G, Notley C, Hackmann C (2018). Experiencing mental health diagnosis: a systematic review of service user, clinician, and carer perspectives across clinical settings. Lancet Psychiatry.

[8] Watts J (2019). Problems with the ICD-11 classification of personality disorder. Lancet Psychiatry.

[9] Chanen AM, McCutcheon LK (2008). Personality disorder in adolescence: the diagnosis that dare not speak its name. Personal Ment Health.

[10] Clark LA, Corona-Espinosa A, Khoo S, Kotelnikova Y, Levin-Aspenson HF, Serapio-García G, et al. Preliminary scales for ICD-11 personality disorder: self and interpersonal dysfunction plus five personality disorder trait domains. Front Psychol. 2021;12 Available from: https://www.frontiersin.org/articles/10.3389/fpsyg.2021.668724/full. 10.3389/fpsyg.2021.668724 PMC8311289 34322060

[11] Spitzer C, Jelinek L, Baumann E, Benecke C, Schmidt AF. Negative self-conscious emotions in women with borderline personality disorder as assessed by an implicit association test. Personal Disord Theory, Res Treat. 2020; Available from: http://doi.apa.org/getdoi.cfm?doi=10.1037/per0000467. 10.1037/per0000467 33211529

[12] Liebke L, Bungert M, Thome J, Hauschild S, Gescher DM, Schmahl C (2017). Loneliness, social networks, and social functioning in borderline personality disorder. Personal Disord Theory, Res Treat.

[13] Staebler K, Helbing E, Rosenbach C, Renneberg B (2011). Rejection sensitivity and borderline personality disorder. Clin Psychol Psychother.

[14] Bateman A, Campbell C, Luyten P, Fonagy P (2018). A mentalization-based approach to common factors in the treatment of borderline personality disorder. Curr Opin Psychol.

[15] Herpertz SC, Huprich SK, Bohus M, Chanen A, Goodman M, Mehlum L (2017). The challenge of transforming the diagnostic system of personality disorders. J Personal Disord.

[16] Storebø OJ, Stoffers-Winterling JM, Völlm BA, Kongerslev MT, Mattivi JT, Jørgensen MS, et al. Psychological therapies for people with borderline personality disorder. Cochrane Database Syst Rev. 2020;5 Available from: http://doi.wiley.com/10.1002/14651858.CD012955.pub2. 10.1002/14651858.CD012955.pub2 PMC7199382 32368793

[17] Stoffers-Winterling JM, Storebø OJ, Völlm BA, Mattivi JT, Nielsen SS, Kielsholm ML, et al. Pharmacological interventions for people with borderline personality disorder. Cochrane Database Syst Rev. 2018; Available from: http://doi.wiley.com/10.1002/14651858.CD012956. 10.1002/14651858.CD012956.pub2 PMC9662763 36375174

[18] Bach B, Sellbom M, Skjernov M, Simonsen E (2018). ICD-11 and DSM-5 personality trait domains capture categorical personality disorders: finding a common ground. Aust New Zeal J Psychiatry.

[19] Hopwood CJ, Kotov R, Krueger RF, Watson D, Widiger TA, Althoff RR (2018). The time has come for dimensional personality disorder diagnosis. Personal Ment Health.

[20] Shedler J, Beck A, Fonagy P, Gabbard GO, Gunderson JG, Kernberg OF (2010). Personality disorders in DSM-5. Am J Psychiatry.

[21] Bach B, Brown TA, Mulder RT, Newton-Howes G, Simonsen E, Sellbom M (2021). Development and initial evaluation of the ICD-11 personality disorder severity scale: PDS-ICD-11. Personal Ment Health.

[22] Oltmanns JR, Widiger TA (2020). The five-factor personality inventory for ICD-11: a facet-level assessment of the ICD-11 trait model. Psychol Assess.

[23] Kim Y-R, Tyrer P, Hwang S. Personality assessment questionnaire for ICD-11 personality trait domains: development and testing. Personal Ment Health. 2020; Available from: https://onlinelibrary.wiley.com/doi/abs/10.1002/pmh.1493. 10.1002/pmh.1493 32638542

[24] Weekers LC, Hutsebaut J, Kamphuis JH (2019). The level of personality functioning scale-brief form 2.0: update of a brief instrument for assessing level of personality functioning. Personal Ment Health.

[25] Gamache D, Savard C, Leclerc P, Payant M, Berthelot N, Côté A, et al. A proposed classification of ICD-11 severity degrees of personality pathology using the self and interpersonal functioning scale. Front Psychiatry. 2021;12 Available from: https://www.frontiersin.org/articles/10.3389/fpsyt.2021.628057/full. 10.3389/fpsyt.2021.628057 PMC8012561 33815167

[26] Goth K, Birkhölzer M, Schmeck K (2018). Assessment of personality functioning in adolescents with the LoPF–Q 12–18 self-report questionnaire. J Pers Assess.

[27] Bender DS, Skodol AE, First MB, Oldham J, First M, Skodol A, Bender D, Oldham J (2018). Module I: structured clinical interview for the level of personality functioning scale. Struct Clin interview DSM-5 Altern model personal Disord.

[28] Hutsebaut J, Weekers LC, Tuin N, Apeldoorn JSP, Bulten E. Assessment of ICD-11 personality disorder severity in forensic patients using the semi-structured interview for personality functioning DSM-5 (STiP-5.1): preliminary findings. Front Psychiatry. 2021;12 Available from: https://www.frontiersin.org/articles/10.3389/fpsyt.2021.617702/full. 10.3389/fpsyt.2021.617702 PMC8085303 33935824

[29] Oltmanns JR, Widiger TA (2018). A self-report measure for the ICD-11 dimensional trait model proposal: the personality inventory for ICD-11. Psychol Assess.

[30] Sellbom M, Solomon-Krakus S, Bach B, Bagby RM (2020). Validation of personality inventory for DSM–5 (PID-5) algorithms to assess ICD-11 personality trait domains in a psychiatric sample. Psychol Assess.

[31] Bach B, Kerber A, Aluja A, Bastiaens T, Keeley JW, Claes L (2020). International assessment of DSM-5 and ICD-11 personality disorder traits: toward a common nosology in DSM-5.1. Psychopathology.

[32] Kerber A, Schultze M, Müller S, Rühling RM, Wright AGC, Spitzer C, et al. Development of a short and ICD-11 compatible measure for DSM-5 maladaptive personality traits using ant Colony optimization algorithms. Assessment. 2020; Available from: http://journals.sagepub.com/doi/10.1177/1073191120971848. 10.1177/1073191120971848 PMC8866743 33371717

[33] Skodol AE, First MB, Bender DS, Oldham JM, Module II, First MB, Skodol AE, Bender DS, Oldham JM (2018). Module II: structured clinical interview for personality traits. Struct Clin interview DSM-5 Altern model personal Disord (SCID-AMPD).

[34] Winsper C, Bilgin A, Thompson A, Marwaha S, Chanen AM, Singh SP (2020). The prevalence of personality disorders in the community: a global systematic review and meta-analysis. Br J Psychiatry.

[35] Kvarstein EH, Antonsen BT, Klungsøyr O, Pedersen G, Wilberg T. Avoidant personality disorder and social functioning: a longitudinal, observational study investigating predictors of change in a clinical sample. Personal Disord Theory, Res Treat. 2021; Available from: http://doi.apa.org/getdoi.cfm?doi=10.1037/per0000471. 10.1037/per0000471 33507789

[36] Fjermestad-Noll J, Ronningstam E, Bach B, Storebø OJOJ, Rosenbaum B, Simonsen E (2020). Psychotherapeutic treatment of depressive symptoms in patients with narcissistic disturbances: a review. J Contemp Psychother.

[37] Watters CA, Bagby RM, Sellbom M (2019). Meta-analysis to derive an empirically based set of personality facet criteria for the alternative DSM-5 model for personality disorders. Personal Disord Theory, Res Treat.

[38] Samuel DB, Widiger TA (2008). Clin Psychol Rev.

[39] Fassbinder E, Arntz A (2018). Schema therapy with emotionally inhibited and fearful patients. J Contemp Psychother.

[40] Simonsen S, Eikenaes IU-M, Bach B, Kvarstein E, Gondan M, Møller SB, et al. Level of alexithymia as a measure of personality dysfunction in avoidant personality disorder. Nord J Psychiatry. 2020:1–9 Available from: https://www.tandfonline.com/doi/full/10.1080/08039488.2020.1841290. 10.1080/08039488.2020.1841290 33146059

[41] Weinbrecht A, Schulze L, Boettcher J, Renneberg B (2016). Avoidant personality disorder: a current review. Curr Psychiatry Rep.

[42] Bach B, Eikenæs IU (2021). Transdiagnostic conceptualization of social avoidance through the lens of personality functioning and traits. J Clin Psychol.

[43] Fjermestad-Noll J, Ronningstam E, Bach B, Rosenbaum B, Simonsen E (2020). Perfectionism, shame, and aggression in depressive patients with narcissistic personality disorder. J Personal Disord.

[44] Hansen SJ, Christensen S, Kongerslev MT, First MB, Widiger TA, Simonsen E (2019). Mental health professionals’ perceived clinical utility of the ICD-10 vs. ICD-11 classification of personality disorders. Personal Ment Health.

[45] Tracy M, Tiliopoulos N, Sharpe L, Bach B (2021). The clinical utility of the ICD-11 classification of personality disorders and related traits: a preliminary scoping review. Aust New Zeal J Psychiatry.

[46] Hackmann C, Balhara YPS, Clayman K, Nemec PB, Notley C, Pike K (2019). Perspectives on ICD-11 to understand and improve mental health diagnosis using expertise by experience (INCLUDE study): an international qualitative study. Lancet Psychiatry.

[47] Bach B, Simonsen S (2021). How does level of personality functioning inform clinical management and treatment? Implications for ICD-11 classification of personality disorder severity. Curr Opin Psychiatry.

[48] Morey LC, Benson KT (2016). Relating DSM-5 section II and section III personality disorder diagnostic classification systems to treatment planning. Compr Psychiatry.

[49] Sharp C, Wright AGC, Fowler JC, Frueh BC, Allen JG, Oldham J (2015). The structure of personality pathology: both general (‘g’) and specific (‘s’) factors?. J Abnorm Psychol.

[50] Wright AGC, Hopwood CJ, Skodol AE, Morey LC (2016). Longitudinal validation of general and specific structural features of personality pathology. J Abnorm Psychol.

[51] Clark LA, Nuzum H, Ro E (2018). Manifestations of personality impairment severity: comorbidity, course/prognosis, psychosocial dysfunction, and ‘borderline’ personality features. Curr Opin Psychol.

[52] Crego C, Widiger TA (2020). The convergent, discriminant, and structural relationship of the DAPP-BQ and SNAP with the ICD-11, DSM–5, and FFM trait models. Psychol Assess.

[53] Sauer-Zavala S, Bentley KH, Wilner JG (2016). Transdiagnostic treatment of borderline personality disorder and comorbid disorders: a clinical replication series. J Pers Disord.

[54] Saulsman LM, Page AC (2004). The five-factor model and personality disorder empirical literature: a meta-analytic review. Clin Psychol Rev.

[55] Kramer U, Kramer U (2019). Individualizing treatment for clients with personality disorders. Case Formul personal Disord tailoring Psychother to Individ client.

[56] Stepp SD, Whalen DJ, Smith TD (2013). Dialectical behavior therapy from the perspective of the five-factor model of personality.

